# Correlations and Kappa Distributions: Numerical Experiment and Physical Understanding

**DOI:** 10.3390/e27040375

**Published:** 2025-03-31

**Authors:** David J. McComas, George Livadiotis, Nicholas V. Sarlis

**Affiliations:** 1 Department of Astrophysical Sciences, Princeton University, Princeton, NJ 08544, USA; dmccomas@princeton.edu (D.J.M.); nsarlis@phys.uoa.gr (N.V.S.); 2 Physics Department, National and Kapodistrian University of Athens, Panepistimiopolis, 15784 Athens, Greece

**Keywords:** space plasmas, solar wind, heliosphere, kappa distributions, correlations, numerical experiment

## Abstract

Kappa distributions, their statistical framework, and their thermodynamic origin describe systems with correlations among their particle energies, residing in stationary states out of classical thermal equilibrium/space plasmas, from solar wind to the outer heliosphere, are such systems. We show how correlations from long-range interactions compete with collisions to define the specific shape of particle velocity distributions, using a simple numerical experiment with collisions and a variable amount of correlation among the particles. When the correlations are turned off, collisions drive any initial distribution to evolve toward equilibrium and a Maxwell–Boltzmann (MB) distribution. However, when some correlation is introduced, the distribution evolves toward a different stationary state defined by a kappa distribution with some finite value of the thermodynamic kappa κ (where κ→∞ corresponds to a MB distribution). Furthermore, the stronger the correlations, the lower the κ value. This simple numerical experiment illuminates the role of correlations in forming stationary state particle distributions, which are described by kappa distributions, as well as the physical interpretation of correlations from long-range interactions and how they are related to the thermodynamic kappa.

## 1. Introduction

Despite the common assumption to the contrary, because of the correlations amongst charged particles, space plasma particle energy distributions are rarely, if ever, characterized by Maxwell–Boltzmann (MB) distributions [1,2]. Instead, kappa distributions with significantly enhanced tails to higher energies are routinely observed in space plasmas. These observations began in the 1960s (see original studies by [3,4,5]) and now extend from the solar wind to planetary magnetospheres, out through the heliosheath beyond the termination shock, and even beyond the heliosphere to interstellar and astrophysical plasmas (Table 1). Kappa distributions are physically well founded and closely connected to non-extensive statistical mechanics, plasma physics, and thermodynamics (e.g., see original papers: [6,7,8,9,10]; books: [11,12,13]; and reviews by [9,14,15,16,17]). These kappa distributions, their statistical framework, and their thermodynamic origin describe systems residing in stationary states out of classical thermal equilibrium (e.g., [9]). Recently, Livadiotis and McComas [18,19] showed that these distributions also constitute the most generalized particle energy distribution function that is consistent with thermodynamics.

The plasma in the heliosheath is especially interesting because its values of kappa are extremely low [88,91] and consistently in what we call the “far-equilibrium” region [80,81]. In particular, since launching in 2008, the Interstellar Boundary Explorer (IBEX) mission [110] has been providing remarkable remote observations of the low thermodynamic kappa characterizing these distant plasmas and how this is related to their strongly correlated particles [80,82,85,88,89,90,91,92,93,98,111]. The Interstellar Mapping and Acceleration Probe (IMAP) mission [112], launching in 2025, will extend these observations with even more precision and spatial resolution.

Classical thermal equilibrium is a stationary state where the particle system (1) has no long-range correlations among its particle energies, (2) entropies are additive (i.e., the entropy of a system is the simple sum of the entropies of its particles or constituent subsystems), and (3) particle velocities or kinetic energies follow an MB distribution, as in Equation (2). Livadiotis and McComas [113] showed that these three properties are equivalent and that any of the three will lead to the other two. Even though classical thermal equilibrium is the basis of the whole framework of classical thermodynamics, it constitutes a rather special and extreme stationary state, which is given by the limit of κ→∞ in Equation (1), which properly defines kappa distributions ([9,11,15,82]; Ch. 4).

The *d*-dimensional kappa velocity u distribution is given by

(1) $$P_{u}\left(u;\kappa_{0}\right)∼{\left[1+\frac{1}{\kappa_{0}}\frac{{\left(u-u_{b}\right)}^{2}}{\theta^{2}}\right]}^{-\kappa_{0}-1-\frac{1}{2}d},P_{u}\left(u;\kappa_{0}\to \infty \right)∼\exp\left[-\frac{{\left(u-u_{b}\right)}^{2}}{\theta^{2}}\right]$$

for a bulk velocity $u_{b}$, thermal speed θ (temperature *T* in speed dimensions) and kappa, $\kappa_{0}$, which parameterize the distribution. The thermodynamic kappa depends on the degrees of freedom *d* in a simple way that $\kappa \left(d\right)=const.+\frac{1}{2}d$, where the constant constitutes the invariant kappa $\kappa_{0}$, which is independent of the degrees of freedom [82,93,114,115]. The limiting case of an MB distribution is obtained for $\kappa_{0}\to \infty$ (or κ→∞). In terms of kinetic energy, $\epsilon =\frac{1}{2}m{\left(u-u_{b}\right)}^{2}$, the distribution in Equation (1) becomes (see Appendix A):

(2) $$P_{E}\left(\epsilon ;\kappa_{0}\right)∼{\left(1+\frac{1}{\kappa_{0}}\frac{\epsilon }{k_{B}T}\right)}^{-\kappa_{0}-1-\frac{1}{2}d},P_{E}\left(\epsilon ;\kappa_{0}\to \infty \right)∼\exp\left(-\frac{\epsilon }{k_{B}T}\right)$$

The kappa distribution is parameterized by the two quantities of temperature and the kappa. While kappa was used simply as a shaping parameter in earlier, observational studies, it is now understood to be a truly physically meaningful quantity that is as fundamental to thermodynamics as the temperature—and so we now commonly refer to it as the thermodynamic kappa to stress this point [85,93,113].

We also now understand that there is a “spectrum-like” arrangement of stationary states, which arise from the framework of generalized thermodynamics [15]. Such systems, residing in any stationary states, are described by kappa distributions and a generalized definition of thermal equilibrium. A system residing in any stationary state other than classical thermal equilibrium (1) has long-range correlations among the system’s particles, (2) is not governed by simple additivity of the particle entropies, and (3) the particle velocities or kinetic energies are distributed according to kappa distributions with κ<∞. Livadiotis and McComas [113] showed that all these three properties are equivalent and that the most generalized unrestricted addition rule of entropies [113,116,117] is a property equivalent to the formulation of Tsallis entropy [19,118,119,120], whose maximization under the canonical ensemble leads to kappa distributions [7,9], a functional form that is consistent with nonzero correlation among particles [11,16,82,115,121,122,123,124]. The crux of all three of these equivalent properties is a revolutionary new concept called the “entropy defect”.

The entropy defect [18,19,85,113,125,126,127,128] describes all of the physically allowed ways that the entropy of a system can be shared among its constituent particles. The total entropy of a system equals the simple sum of the constituent entropies only if there are absolutely no correlations (see also: [7,129,130]). Otherwise, the existence of any correlation adds order to the whole system, and thus decreases its total disorder, or in other words, its entropy. The entropy defect (3) describes this loss of entropy due to the order induced by the presence of correlations caused by long-range interactions among the particles: for two subsystems A and B combined to create the whole system A⊕B, the loss of entropy expressing the entropy defect $S_{D}$ equals the entropy difference of $S_{D}\equiv S_{A}+S_{B}-S_{A⊕B}$. As shown by Livadiotis and McComas [113,117], the most general and physically consistent expression of entropy defect is $S_{D}=\frac{1}{\kappa }S_{A}S_{B}$. Therefore, the partitioning of the total entropy into its parts is simply given by

(3) $$S_{A⊕B}=S_{A}+S_{B}-\frac{1}{\kappa }S_{A}S_{B}.$$

The entropy defect is analogous to the mass defect, which quantifies the missing mass associated with the assembling of subatomic particles, but instead the entropy defect quantifies the missing entropy associated with the assembly of particle systems that have long-range correlations. The entropy defect was developed from first physical principles by Livadiotis and McComas [113,117], and it shows that kappa distributions are precisely formulated with Equations (1) and (2), and as such, constitute the most generalized formalism for describing particle energies that is consistent with thermodynamics ([19,128]; see also: [116,118,129,131,132]). We note that the motivation used by Milovanov and Zelenyi [7] also led them to Equation (3), however using the *q*-notation.

Using the entropy defect, we were able to find the exact entropy of a system of particles with correlations characterized by their energies, are described by a kappa distribution [18,116]. Indeed, the presence of correlations among the particle energies leads a distribution of energies, that once stabilized, is uniquely described by kappa distributions:

-*Kappa Distributions* → *Correlation of Particle Energies*: Given the kappa distributions, we derive the correlation of particle energies. Furthermore, we use the correlations to define the thermodynamic kappa parameter that shapes these distributions, providing its kinetic [82,114,115] and thermodynamic [18] definitions.

-*Correlation of Particle Energies* → *Kappa Distributions*: The existence of particle correlations reduces the entropy of a system [113], and it is this reduction that defines the concept of entropy defect [18]. The formulation of entropy defect is uniquely determined, providing a pseudo-addition rule for entropies [117]. Given this entropy addition, we can derive the expression of entropy in terms of the probability distribution of energy which, as shown, coincides with Tsallis entropy [19,116,119,129]. Maximization of this entropy leads to kappa distributions [6,7,8,9,10,11], whose power-law behavior at high energies is well known [11,27].

In addition, the statistical framework of kappa distribution generalizes the classical formalism of the Sackur [133] and Tetrode [134] entropy,

(4) $$S=\kappa \left[1-{\left(T/T_{0}\right)}^{-\frac{3}{2}\frac{1}{\kappa }}\right],$$

where the thermal constant $T_{0}$ constitutes the minimum temperature for the entropy to be positive; [10,11,31], Chapters 2 and 5.

The formulation of entropy defect, and the respective addition rule of entropies, has surprising similarities to the lattice model [135]. For instance, the Ising model is exactly described by the addition rule of entropy defect where each individual entropy is proportional to the spin, while the kappa parameter (represented by a tensor now) becomes the magnitude of the mutual interactions. Their connection is clear, not only in the MB limit, but also for any kappa. While this has been considered in the past (e.g., [136]), more theoretical effort is needed for improving the understanding of this comparison.

It is worth nothing that, in addition to this fundamental physical understanding from thermodynamics, various microphysical mechanisms have also been shown to generate kappa distributions in space plasmas and other such systems. Some examples are superstatistics (i.e., the temperature is not fixed but has a special distribution; (e.g., [130,137,138,139,140,141,142,143]), shock waves (e.g., [144]), turbulence (e.g., [12,32,45,46,47]), “pump mechanism” acceleration (e.g., [145]), Coulomb interactions (e.g., [146]; see also: [147,148]), colloidal particles [149], and polytropes (e.g., [123,150,151,152,153]). What all of these mechanisms have in common is that they include long-range interactions that generate correlations among the particles and their energies.

We now have a much deeper understanding of the underlying physics of kappa distributions. Any mechanisms associated with long-range interactions induce correlations among particles [154]. The presence of these correlations reduces the value of kappa, which reflects the thermodynamics of the entire distribution [82]. The magnitude of the entropy defect is measured by 1/κ and sets a maximum boundary to the value of entropy [113,117,128]. One use of the entropy defect is to derive entropic equations that lead to transport equations regarding the kappa value of an evolving plasma [85,93]. Even more importantly, when a stationary state finally is achieved from any initially non-stationary particle distribution, it forms a kappa distribution of particle energies that is characterized with the same kappa found through a calculation of the entropy defect [19].

**Figure 1:**
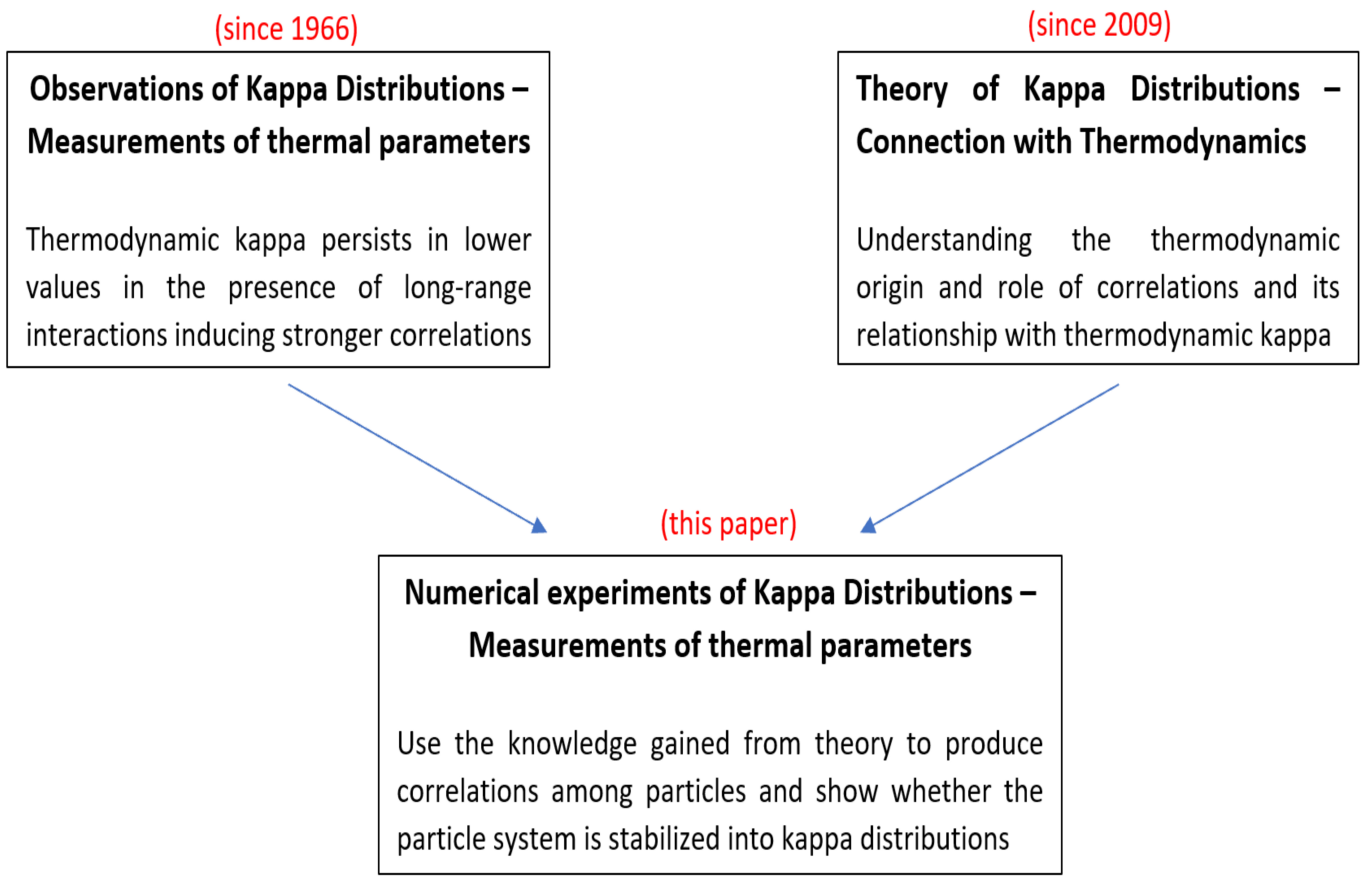
Observations and theory of kappa distributions have been well developed, but what is missing is a simple numerical experiment of the effect of particles correlations on their distributions.

From all of this new knowledge, we ask: Is there yet another, independent way to test our understanding of kappa distributions and their origin in correlations from long-range interactions? What is missing from the above observational and theoretical work is a numerical experiment to test whether a particle energy distribution function, which is described by a MB distribution in the absence of correlations, indeed generates a kappa distribution when correlations are introduced. This would complete the proof of the thermodynamic framework of kappa distributions and their connection with long-range correlations. Such an experiment is exactly the purpose of this paper, as summarized schematically in Figure 1.

Section 2 of this paper describes how we construct and carry out our simple numerical experiment. Results are shown in Section 3 and Section 4 provides our conclusions and discussion. Finally, Appendix A gives the formulation of kappa probability and cumulative distribution function used for describing the distribution of the energies residing in stationary states, while Appendix B provides the simple computer code used for these numerical experiments.

## 2. Method of the Numerical Experiment

For our numerical experiment, we start with a distribution of energies $E_{i}$, where the particles are labeled in sequential position, i=1,…,N. Number *N* is chosen to be large enough to allow for the distribution to evolve into a stationary state with a well-resolved tail (*N*∼$10^{6}$). To emulate collisions, we choose a random particle in the sequence, *i*, which collides with the adjacent particle, i+1, conserving both energy and momentum in the collision. We then randomly choose additional particles (*i*) from the distribution and repeat this procedure many times, until the distributions stop changing, that is, it reaches a stationary state (typically∼$10^{5}$ iterations).

One-dimensional (linear) collisions do not allow for any freedom in the exchange of energies between the colliding particles because conservation of both energy and momentum results simply in the exchange of the particles energies. Here, we include realistic two-dimensional (planar) collisions, which allows for the one degree of freedom that we use as the variable characterizing the ratio of the particle energies after the collision to the (conserved) total energy of the particle pair. Each collision is planar, with the plane determined by the two momentum vectors of the colliding particles. Because the collision plane is different for each of the collisions, this process forms a three-dimensional distribution of particle momenta. Our model does not identify the distance between the particles, so there are only two angular degrees of freedom and not three. Once the distribution reaches a stationary state, the particle energies form a distribution, which is related to the two-dimensional distribution of particle velocities. This particle distribution is recorded and analyzed as an example distribution with only collisional interactions. When there are only collisions (zero correlation to other particles beyond the two involved in each collision), an MB distribution naturally develops, regardless of the initial particle distribution.

Next, we introduce correlations in our numerical experiment. In particular, we add another step after each collision and start by randomly choosing another particle, *j*, somewhere along the position line. We then randomly select between *j* and j+1 to be the “donor” particle, d, and the other to be the “recipient” particle, r. We then take some specified fraction *f* of the energy of the donor (0≤f<1), remove it from the donor, i.e., $E_{d}\to \left(1-f\right)\cdot E_{d}$, and give it to the adjacent recipient, i.e., $E_{r}\to E_{r}+f\cdot E_{d}$, such that energy is conserved. Correlations can be increased in two ways: (1) by increasing the value of the energy transfer fraction *f* in the range [0–1), and (2) by increasing the number of *j*-pairs, *M*, that exchange energies after each *i* collision.

Clearly, both interactions in this simple numerical experiment conserve energy and we argue that both can be thought of as types of collisions. The first type is a typical collision (primary or free collision), characterized by one degree of freedom in the energy of each particle pair, while their combined, total energy is conserved. The second step in our process—the one that produces correlations—we call a “pseudo-collision”, or controlled collision. This collision is also an exchange of energy like a physical collision, but without any degrees of freedom since it is predetermined by our choice of the fraction *f*.

Our invented procedure adds pseudo-collisions in addition to physical collisions. The physical collisions add randomness to the system, while pseudo-collisions do not have randomness and thus increase the correlation of the particles and the persistence of system’s characteristics (e.g., see the persistence vs. randomness that characterize solar energetic protons, as shown in the work of Sarlis et al. [26]). Therefore, our pseudo-collision procedure is a process that should increase the order of the system. This process mimics the mechanism of pickup protons in the heliosphere; for example, where a highly ordered pickup proton charge exchanges and substitutes for a thermally distributed solar wind proton. The pickup process adds order to the solar wind plasma, decreasing its entropy, and thus, driving its thermodynamic kappa to lower values and toward anti-equilibrium [82,83,85,93].

## 3. Results

We construct the complementary cumulative distribution (ccd) of energies, which is the probability distribution of energies greater than a certain energy value *E*, that is, the integration of Equation (2) for energies E≤ϵ<+∞, and then fit it with a two-dimensional kappa distribution function (see Appendix A). The ccd of one particle energy is defined as the probability of the random variable of particle energy to be greater than the value *E*,

(5) $$F_{ccd\kappa }\left(E\right)={\left(1+\frac{1}{\kappa_{0}}\frac{E}{k_{B}T}\right)}^{-\kappa_{0}-1}$$

where the temperature *T* (with $k_{B}$ noting the Boltzmann’s constant), and the thermodynamic kappa parameterize the distribution.

**Figure 2:**
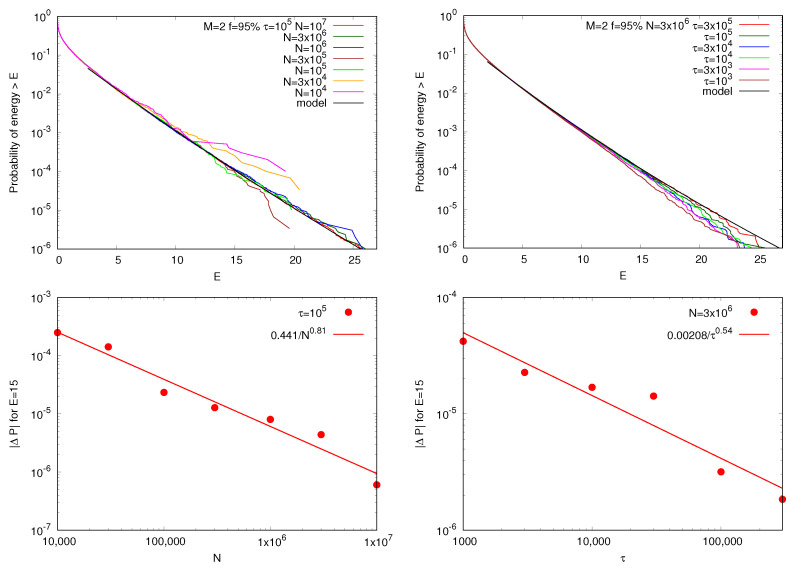
Complementary cumulative distribution (ccd) of particle energies (probability of a particle energy larger than *E*) versus the energy *E*. The simulated distribution converges to a kappa distribution (black) as the number of particles *N* (upper left) and the number of iterations τ (upper right) increase. The lower panels show the respective convergences as *N* and τ increase.

Figure 2 shows that the particle energy distributions for an example (f=95% and M=2) simulation are well described by a kappa distribution, and consequently, the simulated energy ccd distributions are well described by Equation (5). The simulated distributions appear to deviate from a kappa distribution at higher energies, but this is just because of the finite number of particles *N* and iterations τ used in the numerical experiment. As shown in the top panels of Figure 2, as *N* and τ increase, the simulated distributions converge better and better to the modeled kappa distributions. The convergence is quantitively shown in the bottom panels where we define a “distance” measure between each of the converging curves and the converged kappa distributions. We denote this as |ΔP| and plot it for an example energy (here chosen to be at E=15) as a function of the number of particles *N* (left) and iterations τ (right). Both converge increasingly well as *N* and τ increase. Finally, we note that while all these distributions approach a power-law at the limit of high energies (e.g., [27,91]), the fitting is also excellent at lower energies, where the kappa distribution significantly deviates from a power-law.

**Figure 3:**
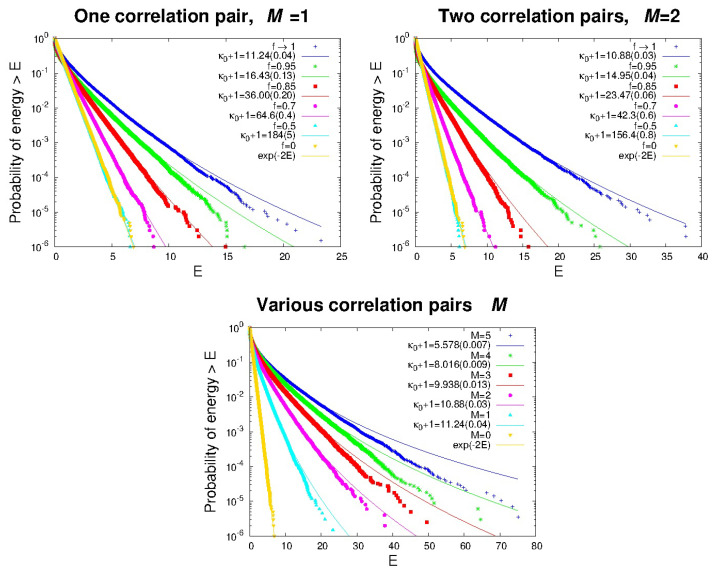
Additional ccd curves for one correlation pair (upper left panel), two correlation pairs (upper right panel), or multiple correlation pairs (lower panel). In the upper two panels, various values of *f* are used while, in the lower panel, the energy transfer fraction is fixed at its maximum, f→1. The deviations of the points below the fit lines at the highest energies are because of the number of particles that it was reasonable to run with our computing resources, but as shown in Figure 2, these converge to the kappa distribution lines with an increasing number of particles or iterations (see also Appendix B). The yellow points (*f* or M=0) correspond to zero correlation and thus MB distributions.

Based on our physical understanding of correlations and long-range interactions, we predict that correlations will drive the distribution function of energies away from a MB distribution and toward a kappa distribution, with more correlation (increasing *f* and/or *M*) producing lower values of the thermodynamic kappa. Figure 3 shows the particles energies that develop a MB distribution (f=0) and other various distributions with f=0.5,0.7,0.85,0.95,∼1. Over many iterations of our procedure, the particle distributions eventually reach a stationary state where the presence of correlations forces the distribution function away from being MB distributed. As the fraction of energy, *f*, transferred between the particles *j* and j+1 and/or the number of additional correlation pairs, *M*, increase, the correlations become increasingly stronger. The larger the correlations, the lower the value of the thermodynamic kappa, and thus, the greater the deviation from the classical MB distribution [15,80,81,82].

We note from the top panels in Figure 3 that, for small *M* (1 and 2) and f→1, the thermodynamic kappa reaches a lower limit of $\kappa_{0}∼10$. Surely, this lower limit does not constitute a physically meaningful value, but instead, simply corresponds to the maximum correlation that can be produced by our simple, invented procedure. Other procedures can lead to stronger correlations, and thus, drive kappa to even smaller values. In the limiting case wherein correlations become so strong that they involve all particles and entirely overwhelm the uncorrelated collisions, thermodynamic kappa approaches its absolute lowest limit, $\kappa_{0}\to 0$. This state is called “anti-equilibrium” due to the total dominance of correlations [15] or the “q-frozen” state, analogous to absolute zero temperature [80,82]. This limiting state is approached in our simple numerical experiment by increasing the number of pairs *M* involved in the transfer of their energies for each single collision. Thus, a combination of larger *f* and *M* values leads to even lower kappa, e.g., for f→1 and M=5, kappa already drops to $\kappa_{0}∼4.6$.

**Figure 4:**
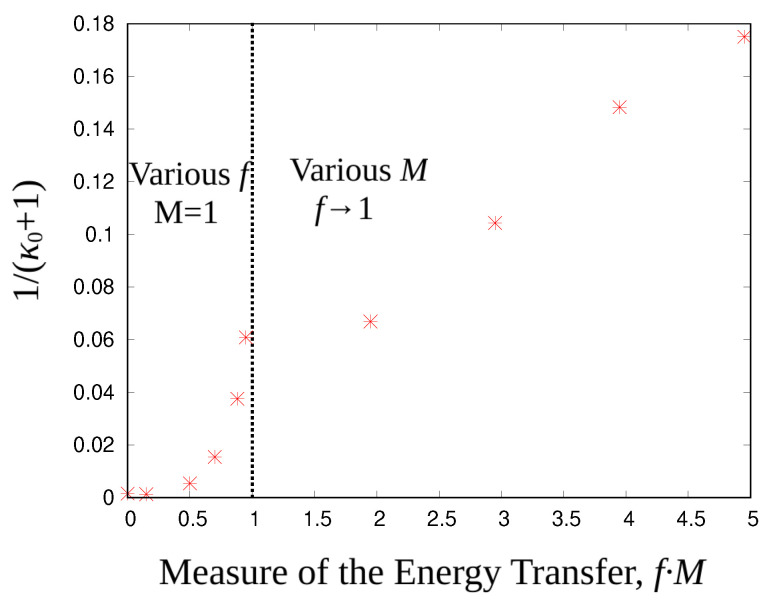
The inverse of $\kappa_{0}+1$ (red asterisks) for the kappa distributions, shown in Figure 3, as a function of f·M (the energy transfer *f* per correlation pair multiplied by the number of correlation pairs *M*).

In order to provide a measure of the correlations caused by the fraction of energy transfer per correlation pair, *f*, and the number of these correlation pairs, *M*, in our invented mechanism we define a measure of the transferred energy through our second pseudo-collision (correlation) procedure as the product of these two factors, f·M. Figure 4 shows the inverse kappa, $1/(\kappa_{0}+1)$ as a function of this transferred energy. Since thermodynamic kappa depends on the dimensionality or degrees of freedom, $\kappa =\kappa_{0}+\frac{1}{2}d$, and given the planar (d= 2) arrangement of each collision, we plot $1/(\kappa_{0}+1)$, instead of $1/\kappa_{0}$ directly. The points with f·M≤1 correspond to one pair (M=1) and various values of *f*, from f=0 (MB distribution) to near its upper limit f→1; points with f·M≥1 correspond to different number *M* pairs, all with maximum f→1 energy transfer. A linear extrapolation indicates that larger values of pairs, *M*∼25, approach the limiting case of $1/(\kappa_{0}+1)\to 1$, or $\kappa_{0}\to 0$, that is, anti-equilibrium [15,82]; however, the functional dependence of $1/(\kappa_{0}+1)$ with f·M is nonlinear and we expect it to asymptotically approach $1/(\kappa_{0}+1)\to 1$ for f·M→N (the total number of particles).

## 4. Conclusions and Discussion

In this paper, we demonstrate for the first time how correlations compete with collisions to generate the specific shape of particle velocity distributions in ways consistent with thermodynamics. Using a simple numerical experiment with unconstrained collisions and additional “pseudo-collisions” with a variable amount of correlation among the particles, we were able to reproduce stationary states out of the classical equilibrium [9,11,15,16,80,81,82]. Systems characterized by particle correlations reside at these generalized thermal equilibria, which are described by the formalism and statistics of kappa distributions; for the special limiting case where there are no correlations among the particles, the system resides at the classical thermal equilibrium, which is described by the classical framework of MB distributions.

In this study, we show the relationship between correlations of particle kinetic energies and thermodynamic kappa using a simple numerical experiment. Here, both randomization and long-range correlations are produced by transferring energy from one particle to another through a sequence of regular collisions and pseudo-collisions (controlled collisions). Random (free) collisions, which are characterized by one degree of freedom in the energy of the two particles, is followed by a number *M* of controlled collisions, which have fixed fraction of energy transfer *f*. This latter procedure produced long-range correlations among the particles in our experiment. Without correlations, collisions drive any initial distribution to evolve toward equilibrium and an MB distribution, that is, a kappa distribution with thermodynamic κ→∞. However, when correlations are introduced, the distribution evolves toward different stationary states defined by kappa distributions with various finite values of kappa κ, depending on the amount of correlation.

The existence of correlations in particle energies means $p_{N=2}\left(E_{i},E_{j}\right)\neq p_{N=1}\left(E_{i}\right)p_{N=1}\left(E_{j}\right)$, where the correlation is measured by the normalized covariance (or otherwise, Pearson’s coefficient) as $\rho \equiv \mathrm{cov}\left(E_{i},E_{j}\right)/\sqrt{\mathrm{var}\left(E_{i}\right)\mathrm{var}\left(E_{j}\right)}$ (e.g., [120,121,122,123,124]). The thermodynamic kappa provides a measure of this correlation of particle energies. In fact, the inverse kappa, 1/κ, is proportional to the correlation coefficient ρ, derived from the distribution of any two particle kinetic energies. The correlation coefficient of kinetic energy, normalized to half the degrees of freedom *d*, equals exactly the inverse kappa, $\rho /(\frac{1}{2}d)=1/\kappa$. This follows a pattern similar to the kinetic definition of the other thermodynamic parameter, the temperature *T*, where the mean kinetic energy *U*, normalized to half the degrees of freedom $\frac{1}{2}d$, equals the temperature, $U/\left(\frac{1}{2}d\right)=k_{B}T$ [11,16,18,82,114,115].

In this study, we have shown the following:

- Stationary states are described by kappa distributions (see also the work of Milovanov et al. [155]).
- Correlations compete with collisions driving the stationary state to lower thermodynamic kappa.
- Inverse thermodynamic kappa describes a measure of correlations.
- Thermodynamic kappa tends to infinity (MB distribution), when no correlations exist.
- Thermodynamic kappa tends to its lowest limit ($\kappa_{0}\to 0$), when the number of correlated particles approaches the whole number of particles.

With these developments, it is now straightforward for researchers to use the already developed, and strongly supported theory of kappa distributions and their genesis in thermodynamics to develop further theoretical concepts, interpret observations via particle correlations and thermodynamics, or even construct more detailed numerical experiments following this analysis.

Thus, this paper provides the critical missing link between observations and theory of space plasma particle distributions. Through a very simple numerical experiment, we demonstrate how the particle energy distribution function evolves from a MB distribution without particle correlations to kappa distributions with decreasing values of thermodynamic kappa through increasing correlations.

## Figures and Tables

**Table 1:** Example observations of kappa distributions ubiquitously in space plasmas.

1. Inner Heliosphere	
solar corona	e.g., [20,21,22]
solar energetic particles	e.g., [23,24,25,26,27,28,29]
solar flares	e.g., [30,31,32,33]
solar radio emission	e.g., [34,35,36]
solar spectra	e.g., [37,38,39]
solar wind	e.g., [40,41,42,43,44,45,46,47,48,49,50,51,52,53,54]
corotating interaction regions	e.g., [55]
**2. Planetary Magnetospheres**	
magnetosheath	e.g., [56,57]
magnetopause	e.g., [58]
magnetotail	e.g., [59]
ring current	e.g., [60]
plasma sheet	e.g., [61,62,63]
magnetospheric substorms	e.g., [64]
aurorae	e.g., [65]
magnetospheres of giant planets, such as:	
- Jovian	e.g., [66,67,68]
- Saturnian	e.g., [69,70,71]
- Uranian	e.g., [72]
magnetospheres of planetary moons, such as:	
- Io	e.g., [73]
- Enceladus	e.g., [74]
cometary magnetospheres	e.g., [75,76]
**3. Outer Heliosphere and Astrophysical Plasmas**	
inner heliosheath	e.g., [77,78,79,80,81,82,83,84,85,86,87,88,89,90,91,92,93,94,95,96,97,98]
H-II regions	e.g., [99]
planetary nebulae	e.g., [100,101,102,103]
active galactic nuclei	e.g., [104,105]
galactic jets	e.g., [106]
supernovae	e.g., [107]
cosmological scale phenomena	e.g., [108,109]

**Figure A1:**
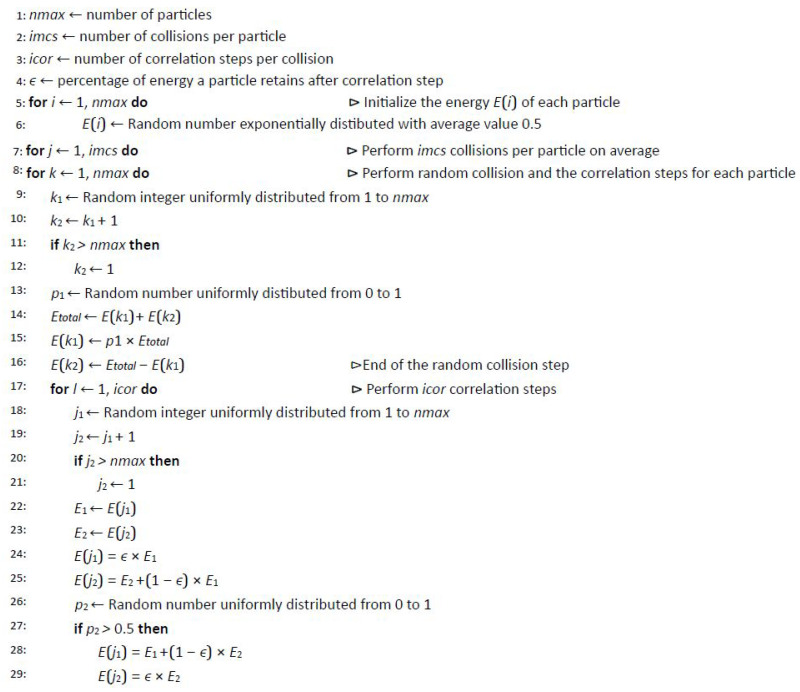
The numerical experiment code used in this study.

## Data Availability

The original contributions presented in this study are included in the article material. Further inquiries can be directed to the corresponding author.

## Appendix A. Formulation of Kappa Distributions

Here, we present the formulation of kappa distributions used in this paper. We investigate a simple model that involves two-dimensional collisions in a system with *N* particles. We retrieve the values of the speeds (magnitudes of the two-dimensional velocities) after the collision and produce their complementary cumulative distribution (ccd) function. Once the distribution resides in a stationary state, it can be modeled by kappa distributions.

The *d*-dimensional kappa distribution velocity distribution is given by

(A1) $$P_{u}\left(u;\kappa_{0}\right)∼{\left[1+\frac{1}{\kappa_{0}}\frac{{\left(u-u_{b}\right)}^{2}}{\theta^{2}}\right]}^{-\kappa_{0}-1-\frac{1}{2}d}$$

for some thermal speed θ (temperature *T* in speed dimensions) and kappa $\kappa_{0}$. The latter constitutes the invariant kappa parameter, a suitable way of expressing thermodynamic kappa when the dimensionality varies. Thermodynamic kappa parameter, κ, depends on the degrees of freedom *d* in a simple way that $\kappa \left(d\right)=const.+\frac{1}{2}d$, where the constant constitutes the invariant kappa $\kappa_{0}$. (For more on the notion and context of temperature and kappa, see: [11,27,80,82,85,88,89,90,91,92,93,115,116,127])

In the case of zero flow speed, we obtain

(A2) $$P_{u}\left(u;\kappa_{0}\right)∼{\left[1+\frac{1}{\kappa_{0}}\frac{u^{2}}{\theta^{2}}\right]}^{-\kappa_{0}-1-\frac{1}{2}d}$$

or, in terms of the kinetic energy $P_{E}\left(\epsilon \right)=P_{u}\left(u=\sqrt{\frac{2}{m}\epsilon }\right)$, i.e.,

(A3) $$P_{E}\left(\epsilon ;\kappa_{0}\right)∼{\left(1+\frac{1}{\kappa_{0}}\frac{\epsilon }{k_{B}T}\right)}^{-\kappa_{0}-1-\frac{1}{2}d}.$$

The density of states for integrating this distribution is $g\left(\epsilon \right)∼\epsilon^{\frac{1}{2}d-1}$. Therefore, the normalization of this distribution is given by

(A4) $$1=\int_{0}^{\infty }P_{E}\left(\epsilon \right)g\left(\epsilon \right)d\epsilon ,$$

or, the corresponding normalization constant is

(A5) $$A=\int_{0}^{\infty }{\left(1+\frac{1}{\kappa_{0}}\frac{\epsilon }{k_{B}T}\right)}^{-\kappa_{0}-1-\frac{1}{2}d}\epsilon^{\frac{1}{2}d-1}d\epsilon .$$

This integration defines the kappa adaptation of the Gamma function (see: Appendix A in Livadiotis and McComas [9]). Moreover, the corresponding energy ccd function is given by the kappa adaptation of the ccd gamma function, which generalizes the classical ccd gamma function, i.e.,

(A6) $$\Gamma_{\kappa }\left(x,d\right)∼\int_{x}^{\infty }P_{E}\left(\tilde{x}\right)g\left(\tilde{x}\right)d\tilde{x}∼\int_{x}^{\infty }{\left(1+\frac{1}{\kappa_{0}}\tilde{x}\right)}^{-\kappa_{0}-1-\frac{1}{2}d}{\tilde{x}}^{\frac{1}{2}d-1}d\tilde{x}.$$

In the two-dimensional case, this becomes

(A7) $$\Gamma_{\kappa }\left(x,2\right)∼\int_{x}^{\infty }P_{E}\left(\tilde{x}\right)g\left(\tilde{x}\right)d\tilde{x}∼\int_{x}^{\infty }{\left(1+\frac{1}{\kappa_{0}}\tilde{x}\right)}^{-\kappa_{0}-2}d\tilde{x},$$

or

(A8) $$\Gamma_{\kappa }\left(\epsilon ,2\right)=\frac{\int_{\epsilon }^{\infty }{\left(1+\frac{1}{\kappa_{0}}\frac{\tilde{\epsilon }}{k_{B}T}\right)}^{-\kappa_{0}-2}d\tilde{\epsilon }}{\int_{0}^{\infty }{\left(1+\frac{1}{\kappa_{0}}\frac{\tilde{\epsilon }}{k_{B}T}\right)}^{-\kappa_{0}-2}d\tilde{\epsilon }},$$

which gives

(A9) $$\Gamma_{\kappa }\left(\epsilon ,2\right)={\left(1+\frac{1}{\kappa_{0}}\frac{\epsilon }{k_{B}T}\right)}^{-\kappa_{0}-1},$$

where this modeled ccd function must be multiplied by the involved number of particles before fitting.

First, we may fit the no-correlation case that leads to MB distribution, in order to find the temperature *T*,

(A10) $$F_{ccd\infty }=\exp\left(-\frac{\epsilon }{k_{B}T}\right),$$

where the ccd refers to one particle energy distribution (it has been normalized to the number of particles).

Next, we proceed to the correlation case, where we expect to have the same parameter value $k_{B}T$. (Nevertheless, the way of inducing the correlations may affect this parameter, namely, a large number of “particles” may have zero speed). Therefore, we choose to fit again the ccd function with two fit parameters, i.e.,

(A11) $$F_{ccd\kappa }\left(\epsilon \right)={\left(1+\frac{1}{\kappa_{0}}\frac{\epsilon }{k_{B}T}\right)}^{-\kappa_{0}-1}.$$

In addition, the two-dimensionality assumption may be suffering from fluctuations of the degrees of freedom, which is called and determined by the phenomenological name of effective dimensionality; here, we also investigate this generalized case of effective dimensionality,

(A12) $$\Gamma_{\kappa }\left(\epsilon ,2\right)=\frac{\int_{\epsilon }^{\infty }{\left(1+\frac{1}{\kappa_{0}}\frac{\tilde{\epsilon }}{k_{B}T}\right)}^{-\kappa_{0}-1-\frac{1}{2}d}d\tilde{\epsilon }}{\int_{0}^{\infty }{\left(1+\frac{1}{\kappa_{0}}\frac{\tilde{\epsilon }}{k_{B}T}\right)}^{-\kappa_{0}-1-\frac{1}{2}d}d\tilde{\epsilon }},$$

leading to the ccd function

(A13) $$F_{ccd\kappa }\left(\epsilon \right)=\frac{A\left(\frac{\epsilon }{\kappa_{0}k_{B}T}\right)}{A(0)},$$

where

(A14) $$A\left(x\right)\equiv \int_{x}^{\infty }{\left(1+\xi \right)}^{-\kappa_{0}-1-\frac{1}{2}d}\xi^{\frac{1}{2}d-1}d\xi .$$

In this paper, we exclusively use Equation (A11), while its generalizetion, Equation (A13), can be used for further future analyses.

## Appendix B. Description of the Code

The computer code used for our numerical experiments is shown in Figure A1. We consider a large number of “particles”, i = 1, 2, …, *N* (we varied *N* from ten thousands up to a million as the variable nmax) of energy $E_{i}$, which are originally distributed according to the Maxwell–Boltzmann (MB) statistics (see line 6 in the algorithm of Figure A1). The specific selection of the original distribution does not affect the final stable distribution, and we have also used uniformly distributed random numbers from 0 to 1 for the initial values of $E_{i}$. We also assume periodic boundary conditions that mean that the “particle” next to the *n*-th particle is the one with i = 1 (see lines 11, 12 and 20, 21 in Figure A1). As mentioned in the main text, we perform the numerical experiment that consist of two steps:

(1) The collision step according to which we randomly select a “particle”, say with $i=k_{1}$, and collide it with the $k_{1}+1$ particle by randomly redistributing their total energy, $E_{\mathrm{total}}=E_{k_{1}}+E_{k_{1}+1}$, among themselves. This means that we select a random number $p_{1}$ uniformly distributed in the region [0, 1] and attribute the new particle energies as, $E_{k_{1}}$ (after collision) $=p_{1}\cdot E_{\mathrm{total}}$ and $E_{k_{1}+1}$ (after collision) $=\left(1-p_{1}\right)\cdot E_{\mathrm{total}}$, (see lines 9–16 in Figure A1).
(2) The pseudo-collision or correlation step where we randomly select a “particle”, say with index $j=j_{1}$, and give its whole energy or a fraction f(=1−ϵ, see lines 18 to 29 in Figure A1) of it to the particle with index $j_{1}+1$. For symmetry reasons, with probability $\frac{1}{2}$ is for the particle with index $j_{1}+1$ that gives its whole energy or a fraction *f* of it to the $j_{1}$ particle. (We note that, for technical reasons, we do not plot the exact case of f=1; this special case corresponds to a standing particle population whose size is governed by nonlinear effects discussed further below).

The correlation step occurs through the pseudo-collisions described in the main text of Section 2. There may be a number *M* of pseudo-collisions per collision step (this corresponds to the integer variable icor in the line 17 of Figure A1); This is important because it increases correlating interactions in the system by choosing additional random indices (*k*, *l*, *m*, etc.) and repeating the second step per each collision step. In our numerical model we use $imcs=10^{5}$ Monte Carlo Steps (MCS), or iterations. For each MCS, steps 1 and 2 are performed *N* times (see line 7 of Figure A1), assigning *N* potentially different energies; thus, MCS mimics the number of particles, *N*.

For large values of the fraction *f*, equilibrium is reached with a significant number of particles at exactly zero energy. This contributes as a delta function to the distribution. The zero energy particles produce nonlinear effects in the modeling and overall different dynamics and statistics. These add non-thermal features to the modeled particles, which are restricted to small energies. The study of these nonlinear effects is beyond the purpose of this simple, first paper, and for the sake of clarity in the figures of this paper, we ignore the particles of zero energy in the calculation of the distributions shown.
